# Positive Arousal Increases Individuals’ Preferences for Risk

**DOI:** 10.3389/fpsyg.2017.02142

**Published:** 2017-12-11

**Authors:** Andrea Galentino, Nicolao Bonini, Lucia Savadori

**Affiliations:** ^1^Department of Psychology and Cognitive Sciences, University of Trento, Trento, Italy; ^2^Fondazione Bruno Kessler, Trento, Italy; ^3^Department of Economics and Management, University of Trento, Trento, Italy

**Keywords:** decision making, risk-taking, risk preferences, positive affect, arousal, positive arousal

## Abstract

Much is known about the effect of negative arousal on decision making, but little is known about the effect of positive arousal. In this study, we manipulated positive arousal and measured individual choices under risk using an incentivized task. Participants were randomly assigned to either a low arousal or a high arousal condition and asked to choose between pairs of two-outcome monetary lotteries with the same expected value but different risk in terms of outcome variance. The probability was set at 50% for each lottery. Participants in the high arousal group selected the riskier lottery more often and took more time to make choices than participants in the low arousal group. This finding shows that introducing a pleasant arousing cue as part of the decision context shifts an individual’s preferences toward the risky economic option and away from the safer one.

## Introduction

A number of studies have examined the effect of negative arousal on the decision making process and its outcome (for a recent review see, Starcke and Brand, 2016), but little attention has been devoted to testing the effect of positive arousal. Positive arousal is defined as the intensity with which pleasure is experienced (Russell, 2003). In comparison with valence, which provides information about the current emotional well-being of the organism (positive vs. negative), arousal refers to the psychological experience of energy, mobilization, activity, tension, alertness, or quietness (Russell and Barrett, 1999). The dimension of arousal ranges from deactivation (calm) to activation (stress or happiness). Arousal is also associated with a bodily experience, because it is characterized by changes in many physiological parameters through the activity of the autonomic nervous system (Hagemann et al., 2003). Arousing reactions, together with pleasantness, provide basic information about the state of the environment and the organism and, most of the time, this information is used as a basis for guiding judgments and decisions as well as for the subsequent cognitive processing (Schwarz and Clore, 1983; Loewenstein et al., 2001; Slovic et al., 2007).

Little is known about the effect of positive arousal on risk preferences, but more is known about the effect of negative arousal on decision making. For example, the effect of stress on decision making has been examined extensively. The studies vary greatly in the type of stress manipulation used and in the type of decision making behavior analyzed. Here, we briefly report only on those studies that measured risk preferences by asking individuals to choose among gambles with equal expected value and that provided individuals with an explicit description of the risk to stay in line with our study’s dependent variable. We exclude those studies that measured decision making under ambiguity (uncertainty). Overall, laboratory findings show that stressed participants are more prone to risky behavior than non-stressed participants. Porcelli and Delgado (2009) first demonstrated that risky gambles became more attractive than equivalent (in expected value) sure gambles when participants were in a stressed state. Other studies employing different sets of lotteries and different types of stress induction found the same results (Lempert et al., 2012; Pighin et al., 2012; Buckert et al., 2014; Richards et al., 2015).

Does positive arousal have the same effect on risk preferences as negative arousal? Few studies have examined the effect of positive arousal (such as, joy, excitement, elation) on risk-taking behavior. In his seminal study, Hirsch (1995) odorized the slot machine area of a Las Vegas casino with a pleasant odor and found that players, as a result, gambled more money. Several subsequent studies examined the influence of positive incidental arousal on risk perception and risk behavior. Ariely and Loewenstein (2006) showed that individuals in a non-aroused state evaluated a series of objects (e.g., women’s shoes) as rather unattractive, but the same individuals in an aroused state (induced by self-stimulation and/or watching sexually arousing stimuli) judged the same objects as much more attractive. In a similar vein, those activities that were originally judged fairly attractive were judged even more attractive under a high-arousal state. These findings show that positive arousal may function as a direct motivator of behavior, increasing the tendency to act unsafely and take risks. These findings also demonstrate that positive arousal “spreads” to unrelated objects or activities, and in doing so might be able to change individual judgments and potentially even choices. Some studies, indeed, have shown that sexual arousal can have an impact on economic behavior (real and hypothetical). McAlvanah (2009) found that individuals revealed an increased preference toward risk in a set of hypothetical gambles after viewing faces of the opposite sex as compared to individuals viewing images of cars. Knutson et al. (2008) demonstrated that males exhibited increased financial risk-taking behavior after viewing erotic images. In a study not investigating economic risk but risk-taking behavior, Dreber et al. (2013) found that male chess players choose significantly riskier strategies when playing against an attractive female opponent. Finally, in a recent finding, Jahedi et al. (2017) demonstrated that males aroused by sexually attractive images were more likely to choose the risky option than the safer option in an incentivized gamble, compared to non-aroused males. However, the risky option in their study was riskier as well as higher in expected value compared to the safe option, making it difficult to disentangle the effect of arousal on risk preferences from the effect of arousal on economic performance (i.e., choosing the best alternative according to the expected value). Positive arousal has also been found to increase suboptimal behaviors, such as impulsive buying (Rook and Gardner, 1993), chocolate consumption (Macht et al., 2002), resistance to temptations (Fedorikhin and Patrick, 2010) and preference for smaller-sooner vs. larger-later reward in intertemporal choice tasks (Wilson and Daly, 2004; Van den Bergh et al., 2008; Kim and Zauberman, 2013).

However, the majority of the studies which show that arousal increases risk-taking behavior employ sexual arousal as the emotional induction (Ariely and Loewenstein, 2006; Knutson et al., 2008; McAlvanah, 2009; Dreber et al., 2013; Jahedi et al., 2017), and many of them were constrained to male-only participants. This, in our opinion, is a limitation of these studies that hampers the generalization of their results to other arousing stimuli that are out of the sexual domain. Moreover, some of these studies used hypothetical choices (McAlvanah, 2009) and not real choices. Here, we present a study that avoids these limitations by arousing participants with a broader set of stimuli that are not confined to the sexual domain and by asking them to make real incentivized economic choices. In doing so, we aimed at broadening the concept of positive arousal to those domains that are common in our everyday decision-making but are not sexually oriented, such as money, sports and social relationships. By adopting this procedure, we also included women in the experimental sample, which had not been done in many previous studies. On this point, it must be said that in some cases stress has been found to affect risk-taking differently depending on gender. Studies have shown that male participants exhibit greater risk-taking behavior under stress whereas females exhibit less risk-taking behavior under stress (Lighthall et al., 2009; Pighin et al., 2015). Thus, we controlled also for the interaction between gender and arousal in investigating whether this moderating effect would be replicated.

Participants in our study were asked to make a series of incentivized economic lottery choices between a risky and a safe option that were identical in expected value. This task enabled us to measure risk-taking in economic decisions. Risk was manipulated by varying the degree of variance between the monetary outcomes of the two lotteries. For example, a lottery that offered a 50% chance of winning $15 and a 50% chance of winning $0 was considered riskier than a lottery that offered a 50% chance of winning $8 and a 50% chance of winning $7, because the outcomes of the first lottery have a greater variance (15 vs. 0) than those of the second one (8 vs. 7). Keeping the expected value of the two lotteries equal (i.e., $7.5) allowed us also to avoid any confounding between the effect of arousal on risk preference and the effect of arousal on economic performance.

In the present study we adopted the technique of contextual priming (Yi, 1990), unlike previous studies that have used classical priming techniques (Knutson et al., 2008; Fedorikhin and Patrick, 2010; Andrade et al., 2016). Classical priming techniques involve a generalized manipulation of arousal, in which participants are induced into an emotional state by viewing arousing images or videos (e.g., McAlvanah, 2009). In contrast, contextual priming requires that a subject is simultaneously exposed to a stimulus (in our case, the gamble) and a contextual factor (in our case, the activating or deactivating images). By adopting the technique of contextual priming (Yi, 1990), we aimed at inducing an association between each lottery and an emotional state, either low or high in arousal, by relating each lottery to a specific image. The simultaneous presentation of the stimulus and the contextual factor creates an association such that the contextual factor can prime certain attributes of the stimulus and influence preferences for choice options (Mandel and Johnson, 2002). The purpose was to observe differences in affect-induced preferences for the same lottery, according to whether this was associated with a high-arousing image or a low-arousing image. This idea stems from studies on affect and the somatic marker hypothesis (Damasio, 1994; Slovic et al., 2004, 2007), where the term ‘affect’ does not denote a state of the individual, but an intrinsic emotional feature of the stimulus, acquired through the repeated association between the stimulus and another stimulus evoking an emotional experience. In the present study, each lottery was associated with either a high or a low arousal image with the aim of assigning an incentive salience to the stimulus (the lottery), and then observing how this impacted the preferences for lotteries that varied in risk.

Participants were assigned to either a high or a low arousal condition and their risk preferences were measured. Previous studies manipulating arousal have adopted mainly two types of research design. The first typically contrasts a high arousal condition with a neutral condition (e.g., Jahedi et al., 2017). For instance, individuals in a high arousal condition view sexual images while those in a neutral condition view pictures of office supplies, tiles or housewares. Such a design enables the researchers to draw conclusions regarding the extent to which the activation of a high arousal state is responsible for the observed deviations of human behavior from a base-line point considered as a neutral or standard conduct. The second type of design usually contrasts a high arousal condition with a low arousal condition, keeping valence constant (e.g., Fernandes et al., 2011). For example, individuals in a high arousal condition view activating images of money or exciting sports while those in a low arousal condition view deactivating images of soft white rabbits or relaxing landscapes. Such a design allows researchers to evaluate the influence of arousal, regardless of valence. More precisely, researchers can exclude the possibility that the resulting behavioral differences observed between individuals induced in a high versus a low arousal state are due to differences in valence and not to differences in arousal. In the design, indeed, valence is kept constant between conditions (i.e., it is always positive). However, whereas the first type of design cannot exclude such a confounding, the second type of design does not allow researchers to determine whether the manipulation influences behavior by facilitating, or inhibiting a process compared to a base-line condition. In the present study, we decided to adopt the second type of design and to keep valence constant in order to reach conclusions that avoid a possible confounding between arousal and valence.

The effect of positive arousal on risk preferences has been explained in various ways. It has been suggested that positive arousal might increase anticipatory affect, thus increasing the desire for reward (Knutson et al., 2008) and the preference for immediate, compared to future, reward (Van den Bergh et al., 2008). The effect of opposite-sex faces on gambling has also been attributed to the activation of a mating mindset or to an increase in competitiveness (McAlvanah, 2009). More importantly, several authors have demonstrated that positive arousal causes a state of cognitive depletion in which the individual’s attention is focused on a very specific aspect of the situation (e.g., the reward or the penalties), thus altering choice behavior (Rook and Gardner, 1993; Ariely and Loewenstein, 2006; Fedorikhin and Patrick, 2010; Starcke and Brand, 2012; Laier et al., 2014; Skakoon-Sparling et al., 2016). According to these explanations, we can make a prediction regarding an individual’s preference for risk under arousal. Let’s consider a choice between two alternatives, one of which is riskier than the other. The riskier alternative is usually associated with higher gains; thus a focused attention on the reward would increase an individual’s preference for the riskier lottery. The riskier alternative, however, is also associated with higher losses; thus, a reduction in attention on the losses would seemingly increase the individual’s preference for the riskier lottery. In considering the extent to which an individual’s preference is influenced by the amount of attention paid to the different aspects of the choice scenario, the assumptions of the cognitive depletion hypothesis suggest that positive arousal should impact an individual’s choice under risk, increasing risk-taking and increasing decision time. Therefore, we expect that participants in the high arousal condition will choose the riskier option more often than those in the low arousal condition and that they will take more time to make their decision.

## Materials and Methods

### Participants

The participants in the study were 126 undergraduate students (*M*_age_ = 22.74 years; 64 females)^1^. Students were recruited through a campus email announcement promising monetary reward for participation in a decision making task. Eligibility criteria were defined as follows: (i) being in good health; (ii) not having actual or previous episodes of psychopathology; and (iii) not being under psychopharmacological treatment. Before confirming their participation in the study all participants were asked to carefully read an information sheet describing the aim of the study, the eligibility criteria, the experimental procedure, and the remuneration procedure. The study’s protocol was approved by the University Ethics Committee for Experimentation on the Human Being of the University of Trento. The study was carried out in accordance with the recommendations of the University Ethics Committee and written informed consent was acquired from all the participants prior to attendance.

### Design

Arousal (high vs. low) was manipulated in a between-subjects design. Participants were randomly assigned to one of two experimental conditions: the high arousal and the low arousal condition (high arousal, *n* = 65; *M*_age_ = 22.94; 32 females; low arousal, *n* = 61; *M*_age_ = 22.53; 32 females).

### Materials and Procedure

The experiment was conducted at the University Experimental Economic Laboratory, in a large room with 24 carrels divided by partitions that prevent visual contact and discourage conversation with neighbors. On arrival at the lab participants drew numbers randomly to learn their assigned carrel and were asked to observe silence. Participants were told that they would complete two tasks: the risk-taking task and the affective experience task. All the tasks were run on PCs (Windows *7*, Intel processor) and presented on monitors with a 1920 × 1080 resolution. The task was developed using the Borland Delphi software package. Participants first read the instructions on the screen under the guide of the experimenter; then the lights of the laboratory were turned off to encourage individual attention and the experiment began with a practice trial of the risk-taking task.

#### Risk-Taking Task

Risk-taking was assessed by asking participants to choose between pairs of two-outcome lotteries presented in 24 trials: 18 were experimental trials and six were filler trials. The order of presentation of the 24 trials was randomized between participants. **Table 1** lists the 18 experimental trials used in this study. In each experimental trial we presented participants with a pair of two-outcome lotteries, A and B, which shared the same expected value but differed in terms of risk. Filler trials were included to ensure that participants did not choose randomly. The filler trials consisted of six choices between pairs of two-outcome lotteries that differed in probability of occurrence and expected value. Participants who did not prefer the dominant option in at least five out of six filler trials were excluded from the analyses.

**Table 1:** Pairs of two-outcome lotteries used in the Risk-Taking Task.

	Riskier lottery		Safer lottery		
Trial #	Outcome A	Outcome B	Outcome A	Outcome B	Expected value (EV)
1	Winning €11	Winning €0	Winning €6	Winning €5	5.5
2	Winning €12	Winning €0	Winning €7	Winning €5	6
3	Winning €13	Winning €0	Winning €6	Winning €7	6.5
4	Winning €15	Winning €0	Winning €8	Winning €7	7.5
5	Winning €16	Winning €0	Winning €7	Winning €6	8
6	Winning €20	Winning €0	Winning €11	Winning €9	10
7	Winning €6	Losing €1	Winning €2	Winning €3	2.5
8	Winning €7	Losing €2	Winning €3	Winning €2	2.5
9	Winning €11	Losing €3	Winning €5	Winning €3	4
10	Winning €10	Losing €1	Winning €5	Winning €4	4.5
11	Winning €11	Losing €1	Winning €6	Winning €4	5
12	Winning €14	Losing €1	Winning €7	Winning €6	6.5
13	Winning €16	Losing €2	Winning €8	Winning €6	7
14	Winning €16	Losing €1	Winning €8	Winning €7	7.5
15	Winning €18	Losing €3	Winning €8	Winning €7	7.5
16	Winning €18	Losing €2	Winning €9	Winning €7	8
17	Winning €19	Losing €3	Winning €9	Winning €7	8
18	Winning €33	Losing €3	Winning €14	Winning €16	15

*For each monetary outcome, probability of occurrence was fixed at 50%. In addition to the 18 stimuli listed above, the following filler trials (pairs of two-outcome lotteries with different probability and different expected value) were included. Filler #1: 60% chance of winning €4 or 40% chance of winning €3 [EV = 3.6]; 70% chance of winning €1 or 30% chance of winning €2 [EV = 1.3]; Filler #2: 60% chance of winning €5 or 40% chance of winning €2 [EV = 3.8]; 30% chance of winning €3 or 40% chance of winning €1 [EV = 2.2]; Filler #3: 30% chance of winning €5 or 70% chance of winning €3 [EV = 3.6]; 20% chance of winning €8 or 80% chance of winning €2 [EV = 3.2]; Filler #4: 70% chance of winning €6 or 30% chance of winning €1 [EV = 4.5]; 40% chance of winning €4 or 60% chance of winning €2 [EV = 2.8]; Filler #5: 60% chance of winning €7 or 40% chance of winning €2 [EV = 5]; 20% chance of winning €4 or 80% chance of winning €3 [EV = 3.2]; Filler #6: 60% chance of winning €10 or 40% chance of winning €1 [EV = 6.4]; 80% chance of winning €2 or 20% chance of winning €1 [EV = 1.8]. Under this condition (of different expected value) participants are expected to prefer the lottery with the higher expected value.*

**FIGURE 1:**
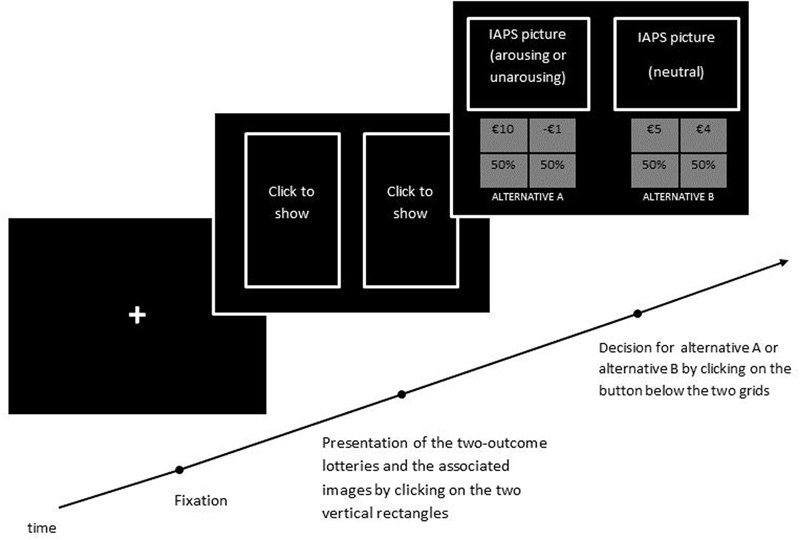
Time course of one trial of the Risk-Taking Task.

The 18 experimental trials presented lotteries that offered the participant the opportunity to win or lose a monetary reward with a 50% probability. For example, lottery A offered a 50% chance of winning €7 or a 50% chance of winning €5 and Lottery B offered a 50% chance of winning €12 or a 50% chance of winning €0. The two lotteries were displayed in two four-cell grids each with the two monetary outcomes displayed in the two upper cells and the probability in the two lower cells (see **Figure 1**). Among the set of risky lotteries, six included a zero gain as outcome (e.g., €12, 0.5; €0, 0.5) and 12 included a loss as outcome (e.g., €10, 0.5; €-1, 0.5). It must be noted that our list of lottery choices does not allow us to measure individual risk aversion because that was not the aim of the study. Our aim was to measure risk-taking as inferred by the decision maker’s actual choice made between two options, one riskier than the other. We used a well-established definition of risk as “outcome variance” where the riskier of two lotteries with the same expected value is that with higher outcome variance (Weber et al., 2004). Similar lottery choices have been used to measure risk-taking in previous studies (e.g., Schmidt et al., 2013).

#### Affective Induction

Affect was induced by having the participants view affective images. We chose the images from the International Affective Picture System (IAPS; Lang et al., 2005) and selected them according to the affective norms^2^. The stimulus set comprised 24 pleasant, high-arousing, 24 pleasant, low-arousing, and 24 neutral color images. The pleasant, high-arousing picture set included images of people having fun, playing extreme sports and erotic stimuli. The pleasant, low-arousing picture set included images of landscapes, flowers, scenes from outer space, cute animals, and serene faces. The neutral picture set included images of objects and geometric shapes. High arousing and low arousing images differed considerably from each other in IAPS normative arousal ratings (*M* = 6.43 and 3.20) but not in valence ratings (*M* = 6.91 and 6.17). Pleasant and neutral images differed from each other in IAPS normative valence ratings (*M* = 6.54 and 4.99) and in arousal ratings (*M* = 4.82 and 2.67).

In order to induce a high and a low arousal, the images were associated with the two-outcome lotteries so that each lottery of the pair was associated with an image that could either activate or deactivate the individual, depending on which condition the participant belonged to. At the beginning of each trial a fixation cross was displayed for a random interval between 100 and 300 ms (see **Figure 1**). Next, two vertical rectangles with the label “click to show” were displayed. Participants had to click with the mouse on each rectangle in order to reveal the pair of two-outcome lotteries and the associated images. To induce an affective state during choice, the affective manipulation (i.e., the image) and the stimuli (i.e., the lottery) were revealed in exactly the same moment. One image was neutral and the other was high (or low) in arousal. The association between the lottery and the arousing (not arousing) image was counterbalanced across participants, so that for each individual who saw the arousing (not arousing) image associated with the riskier lottery, another saw the arousing (not arousing) image associated with the safer lottery. The other lottery was always associated with a neutral image. The left/right presentation of the riskier and safer lottery was also randomized, so that in some trials lottery A was the safer option and in other trials lottery B was the safer option. Each grid containing the lottery was placed inside the image in the lower part, with a button reporting the label “Alternative A” or “Alternative B” below it. Only after revealing both lottery A (with its associated image) and lottery B (with its associated image) could participants select the lottery they preferred by clicking on the respective button. There was no time limit to providing an answer. When a decision was made, participants moved to the next trial. In order to avoid interfering with participants’ affective states, no feedback was provided after a choice was made. In addition to participants’ preferences for lottery A or lottery B, the decision time for each choice was also measured.

#### Manipulation Check

After completing the risk-taking task, participants were presented with the affective experience task. In this task, they saw all the previously seen pictures and reported their current affective state using SAM (Self-Assessment Manikin) scales. Following Lang et al. (2005), we used a computerized version of the two nine-point SAM scales which asks participants to rate their level of experienced valence and arousal while viewing each image previously seen in their specific experimental condition.

#### Post-task Questionnaire

As a last task, participants were asked to provide information about their age, gender, and education level.

#### Payment Scheme

Participants were told initially that they already gained a €3 participation fee for taking part in the study. Furthermore, they were told and reminded throughout that one pair of gambles would be selected at random at the end of the experiment and that the lottery they had chosen from that pair would be played for real money. After they had completed the task, the computer determined which of their choices would be played for real, and then played the lottery to determine the outcome of the gamble they had chosen. In case of loss, the corresponding amount was subtracted from the €3 participation fee. For this reason, negative payoffs did not exceed €3 (see **Table 1**). At the end of the experiment, after providing arousal and valence ratings for each image, participants saw on the computer the trial extracted for their remuneration, their choice in that trial, and the corresponding outcome. They were then paid in cash.

### Statistical Analysis

We used independent samples *t*-tests to measure the size of the difference between the mean valence and arousal ratings for the high arousal images versus the low arousal images. Independent samples *t*-tests were also used to check for differences in mean valence and arousal ratings between males and females and to check for differences in decision times between conditions. Paired samples *t*-test were used to test for any difference regarding both valence and arousal between emotional-content and neutral photographs. To test our main assumption regarding the effect of arousal on risk preferences, we developed a generalized linear mixed model of logistic regression including arousal, gender, and the interaction between the two as fixed effects, and the intercept estimated for each participant as random effect, indicating the participant identification variable as a cluster, as required by the mixed models procedure. The choice made by participants across trials was used as the dichotomous dependent variable. The safer lottery was coded as “0” and the riskier lottery as “1.” A generalized linear mixed model was used because the responses to the 18 trials were not independent. Because each participant contributed to many data points (the 18 trials), these data are not independent as they come from the same participant. In order to analyze these data appropriately, generalized mixed models take this non-independence into account by adding random effects. Decision time and valence were also included in the model as a covariate to check for any effect. Filler trials were excluded from the analysis and only the choices made through the 18 experimental trials were analyzed. Statistical analyses were carried out using the SPSS statistical software package (IBM SPSS version 24).

## Results

One participant was excluded for not having chosen the dominant option in at least five filler trials in the risk-taking task, so that the final sample resulted in 125 participants (high arousal: *n* = 65; 32 females; low arousal: *n* = 60; 31 females).

### Affective Experience Task

Results showed the expected significant difference between the self-reported arousal ratings evoked by the images in the high and low arousal conditions: ratings by participants in the high arousal condition were higher than those expressed by participants assigned to the low arousal condition, attesting that the manipulation was effective *t*(123) = 5.62, *p* < 0.0001, *d* = 1.01 (high arousal: *M* = 5.76, *SD* = 1.56; low arousal: *M* = 4.32, *SD* = 1.25). In addition, participants in the high arousal condition reported higher levels of valence in response to emotional stimuli than did participants in the low arousal condition *t*(123) = 2.43, *p* = 0.01, *d* = 0.43 (high arousal, *M* = 6.98, *SD* = 0.97; low arousal, *M* = 6.6, *SD* = 0.73). However, participants in both groups reported a mean level of valence above the neutral midpoint of the scale (5) indicating that they both experienced positive affect. Moreover, the emotional-content photographs were rated higher in both valence, *t*(124) = 19.78, *p* < 0.0001 (emotional: *M* = 6.80, *SD* = 0.89; neutral: *M* = 4.90, *SD* = 0.70), and arousal ratings, *t*(124) = 13.11, *p* < 0.0001 (emotional, *M* = 5.07, *SD* = 1.59; neutral, *M* = 2.87, *SD* = 1.39) compared to neutral ones. Female participants reported higher ratings of valence and arousal than males [valence: *t*(123) = -2.61, *p* = 0.01, *d* = 0.47; males, *M* = 6.59, *SD* = 0.90; females, *M* = 7.00, *SD* = 0.82; arousal: *t*(123) = -1.98, *p* < 0.05, *d* = 0.35; males, *M* = 4.79, *SD* = 1.42; females, *M* = 5.34, *SD* = 1.7].

### Effect of High vs. Low Arousal on Risk Preference

**FIGURE 2:**
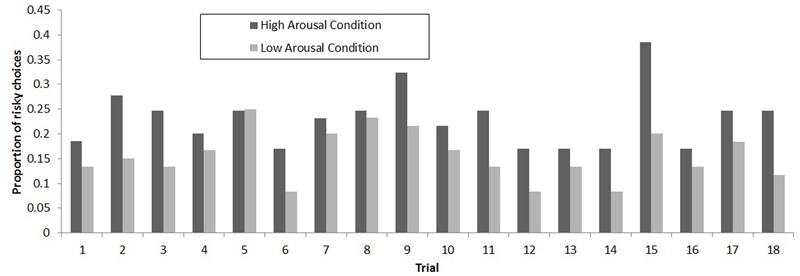
Proportion of risky choices for each trial in the high arousal (*n* = 65) and low arousal (*n* = 60) conditions. Proportions were calculated as the number of choices of the risky lottery summed across participants, divided by the number of participants in that condition.

Analyses revealed a significant main effect of arousal on predicting risky choices, *F*(1,2245) = 4.47, *p* = 0.03. Participants in the high arousal condition selected the riskier option more often than did participants in the low arousal condition (high arousal: *M* = 4.14, *SD* = 3.9; low arousal: *M* = 2.8, *SD* = 3.38)^3^^,^^4^ (see **Figure 2**). A main effect of gender was also found, *F*(1,2245) = 3.7, *p* < 0.05. Males made more risky choices than females (males: *M* = 4.15, *SD* = 3.91; females: *M* = 2.86, *SD* = 3.39). Finally, the interaction effect between arousal and gender was not significant in predicting risky choice (*p* = 0.38).

Ratings provided during the affective experience task revealed that the high and low arousal groups differed in the experienced arousal, confirming that the arousal manipulation was effective. However, they also differed in valence. For this reason, we performed the regression model again introducing valence ratings as a covariate. Valence ratings did not influence risky choice directly (*p =* 1) and the effect of arousal on risk preference was still significant, *F*(1,2194) = 4.51, *p* = 0.03.

An independent samples *t*-test also revealed that participants in the high arousal condition took on average more time than participants in the low arousal condition to make each choice, *t*(123) = 2.46, *p* = 0.01, *d* = 0.44 (high arousal: *M* = 10269.16, *SD* = 4071.01; low arousal: *M* = 8517.33, *SD* = 3841.86). Including the decision time measure into the regression model as a covariate showed that it significantly predicted risky choice, *F*(1,2242) = 19.82, *p* < 0.001, and eliminated the effect of condition, *F*(1,2242) = 3.48, *p* = 0.06. Summarizing, participants assigned to a high arousal condition made more risky choices and took more time to decide than those assigned to a low arousal condition.

## Discussion

By adopting the technique of contextual priming (Yi, 1990), we aimed to investigate the effect of positive arousal on risk preferences. We found that introducing a pleasant arousing cue as part of the decision context increases individual preferences for the risky option, a result that is in line with our hypothesis. Our findings are consistent with those of a growing literature on arousing effects on risk propensity, which finds that positive arousal and risk-taking behavior are positively correlated (e.g., McAlvanah, 2009; Laier et al., 2014).

In our work the two experimental groups differed in the level of experienced arousal as indicated by the self-reported indices of arousal provided in the affective experience task. However, they also differed in terms of valence: the high arousal group reported higher positive valence than the low arousal group. This is not an unexpected result because stimuli rated as more pleasant are rated also as more arousing (Bradley and Lang, 2007). Moreover after controlling for valence, the effect of arousal on risky choice was still significant. Therefore, we can conclude that differences in risky choices found between the two experimental groups are not better explained by differences in valence ratings.

Why does arousal increase risk-taking? Existing explanations refer to a cognitive depletion mechanism (i.e., reduced cognitive resources) induced by arousal (Rook and Gardner, 1993; Ariely and Loewenstein, 2006; Fedorikhin and Patrick, 2010; Laier et al., 2014; Skakoon-Sparling et al., 2016). Support for this explanation is provided by findings that show that a reduction in cognitive capacity is accompanied by an altered sensitivity to reward and immediate gratification, which triggers increased risk-taking (Venkatraman et al., 2011; Ferrara et al., 2015). Further support is also provided by findings that show that positive arousal is linked to an increase in anticipatory affect, which increases the desire for reward (Knutson et al., 2008). As greater risk is often associated with greater reward, an altered sensitivity to reward may explain why arousal increases risk-taking. However, as pointed out in the introduction, risk is associated with higher gains but also with higher losses; hence a greater preference for risk might also be the result of a decreased sensitivity to losses. In the present study we can reasonably rule out a third hypothesis; that is, that arousal alters the sensitivity to the chances (probability) of winning or losing, as in our design the probability was set at the 50% level for both the risky and the safe lottery.

Why does arousal increase sensitivity to potential reward? One explanation refers to a bias in attention. Arousal theories correlate the arousal level of emotional stimuli to attention, in the sense that high-arousing stimuli are capable of capturing attention (Fernandes et al., 2011). Lang et al. (1993) demonstrated that participants look at arousing images for longer than non-arousing images regardless of valence. We found indirect evidence of this in our findings: the high arousal group showed longer decision times presumably because participants spent more time looking at the arousing stimuli. Indeed, when controlling for decision times the effect of arousal on choice disappeared. This could be interpreted as evidence that arousal influenced risky choice by increasing decision time. However, more research is needed to support this explanation. Whilst we lack direct evidence, we believe that arousal may have interfered with the elaboration of some characteristics of the gamble. Skin conductance, a feature of arousal, has been found to be correlated with the interference effect on an emotional Stroop task (Gronau et al., 2003). In a related study, participants were asked to ignore emotional stimuli (IAPS pictures) while performing both a cognitive task (solving math problems) and an attentional task (detecting the location of a line). It was found that the arousal level of the pictures predicted the interference effect on both tasks: the more arousing were the pictures (irrespective of valence) the strongest the interference effect (Schimmack, 2005).

One potential explanation for our findings, therefore, refers to the fact that arousing images might have captured the participant’s attention, thereby leaving few cognitive resources for the thoughtful elaboration of other features of the decision, such as the amount of gains and losses. It has been found that higher levels of arousal increase judgments of probability (Vosgerau, 2010), but in the current study the probability was set at the 50% level for both the safe and the risky lottery; therefore, an alteration in probability estimation cannot account for the effect. Rather, a selective reduction of the attention devoted to losses might more reasonably explain our findings.

## Conclusion

In this study we conducted an experimental manipulation of positive arousal to investigate its effect on risk preferences. Consistent with the cognitive depletion hypothesis, participants were more likely to choose a risky prospect when a high-arousing image was inserted in the decisional context than when a low arousing image was inserted in the context. Also, consistent with this hypothesis, decision times were longer in the high arousal condition than in the low arousal condition.

The insertion of an arousing cue in the decisional context has a long tradition in the field of advertising and marketing. Thus our findings can have implications for researchers and operators in this field. The contextual priming procedure adopted in this study shows that the context can shape individual preference, presumably by activating particular attributes of the choice options. Advertising, for example, aims at associating a positive emotion with an attribute of the product through contextual priming (e.g., an arousing stimuli presented together with a product enhances its positive attributes). However, few studies have addressed choices under risk. This study shows that an arousing cue increases preferences for the riskier alternative. Our findings predict that sales of risky products (e.g., unhealthy food, online shopping, new products, risky investments, etc.) will increase if they themselves prompt, or the context prompts, a high positive arousal.

The present study suffers from a number of limitations. The first weakness is that it does not include a neutral condition, but contrasts only high-aroused individuals with low-aroused individuals. Including a neutral condition could have helped the interpretation of the results, in particular by establishing a baseline risk-taking point. The low arousal condition cannot be considered as equivalent to a neutral condition, and there is therefore a need for future research to include a neutral condition in the design. Another weakness is the lack of any direct evidence in support of the hypothesis that the observed effect is based on attention allocation. Future studies involving methodologies for attention allocation detection, such as some process-tracing measures (e.g., eye-tracking), might help to identify the attentional mechanisms that determine the impact of arousal on choice by directly testing the amount of attention allocated toward each aspect of the gamble in presence of a contextual arousing cue. In particular, as it seems very likely that arousal has the effect of reducing sensitivity to losses, future research should be devoted to measuring the amount of attention allocated toward potential losses. In this experiment, the probability was kept constant at the 50% level for both the risky and the safe alternative; future studies could also investigate whether the effect of positive arousal on risk-taking is sensitive to variations in the probability of the outcomes.

## Author Contributions

AG, NB, and LS designed the research, analyzed the data, interpreted the data, and wrote the paper. AG collected the data.

## Conflict of Interest Statement

The authors declare that the research was conducted in the absence of any commercial or financial relationships that could be construed as a potential conflict of interest.

## Footnotes

1 To estimate the appropriate sample size, we ran an *a priori* sample size calculation using the software package *G*^∗^power (Faul et al., 2007). We used the following settings: effect size *f* = 0.25 (medium effect), *α* err prob = 0.05 (traditional criterion of statistical significance), power = 0.80, numerator *df* = 1 (number of levels: -1) number of groups = 2 (high arousal vs. low arousal). The total sample size suitable to detect effects of a between-participants effect under these conditions would be 128.

2 List of IAPS pictures used in this experiment. *High arousal*: 2344; 2352; 4652; 4653; 4656; 4658; 4659; 4660; 4664; 4670; 4681; 4683; 4695; 4800; 4810; 5629; 8030; 8191; 8210; 8300; 8370; 8400; 8490; 8501. *Low arousal*: 2397; 2514; 2580; 2850; 5000; 5020; 5030; 5220; 5250; 5300; 5500; 5520; 5631; 5635; 5720; 5731; 5764; 5779; 5780; 5891; 7140; 7180; 7490; 7900. *Neutral*: 7000; 7002; 7004; 7006; 7009; 7010; 7020; 7025; 7035; 7040; 7041; 7050; 7052; 7053; 7055; 7056; 7056; 7057; 7059; 7080; 7090; 7170; 7175; 7185; 7186.

3 Because the erotic IAPS pictures were 50% of the high arousal stimuli, we checked statistically whether risk preference was independent from the type of picture presented (erotic vs. non-erotic). In order to do this, we created a dummy variable specifying the type of picture (erotic or no erotic). We performed a generalized linear mixed model including the dummy as a fixed effect. No significant effect of the type of picture on risk preference was found (*p* = 0.15).

4 To estimate power for the predictor variable main effect (to see if there is a difference in participant’s choices of the risky prospect based on condition) we ran a *post hoc* power analysis calculation using the software package *G*^∗^power (Faul et al., 2007). Effect size was calculated by subtracting the mean for the aroused group – mean of the non-aroused group and dividing by the standard deviation of the non-aroused group which gave an effect size of (4.14–2.8)/3.38 = 0.39. We used the following settings: effect size f = 0.39, *α* err prob = 0.05 (traditional criterion of statistical significance), total sample size = 126, numerator df = 1 (number of levels: -1) number of groups = 2 (aroused vs. non-aroused). The resulting power was = 0.9914287.
